# Administrative management and service quality in the dental offices within the context of an upper middle-income country

**DOI:** 10.1371/journal.pone.0307773

**Published:** 2024-09-11

**Authors:** Miriam Maribel Ramírez Altamirano, Luis Alexander Orrego-Ferreyros

**Affiliations:** Facultad de Ciencias de la Salud, Escuela de Estomatología, Universidad César Vallejo, Piura, Perú; University of Maribor, SLOVENIA

## Abstract

This study examined the correlation between administrative management and service quality in dental offices in an upper middle-income country. An applied research approach with an observational, cross-sectional, and exploratory design was used. The sample included 100 patients aged 18 and older from dental offices in Peru, during 2023. Convenience sampling was employed. Spearman correlation and linear regression analyses were conducted. Results showed a positive and moderately strong correlation between administrative management and service quality (Spearman’s rho = 0.79, p < 0.001). Effective organizing (β = 2.11, p < 0.001) and controlling (β = 1.58, p = 0.034) in administrative management were significantly associated with improved service quality. The study concludes that better administrative management positively impacts the quality of service in dental offices.

## 1. Introduction

Service quality is defined as the ability of an organization to meet or exceed the expectations of its customers. The quality of health services, including dentistry, has become increasingly relevant worldwide, becoming a key indicator of the well-being and efficiency of health systems [1, 2]. Management is an activity concerned with guiding human and physical resources such that organizational goals can be achieved [3]. In the global landscape, it is widely recognized that competent and efficient administrative management is crucial for the continuous improvement of the quality of these services [4]. This management not only impacts operational efficiency and patient satisfaction, but also influences treatment effectiveness and patient safety [5, 6]. In Latin America, this trend is of particular interest. The region, with its unique challenges in terms of socioeconomic diversity and disparities in access to healthcare, has begun to recognize the critical importance of administrative management in healthcare delivery [7, 8]. This is especially relevant in the dental sector, which often faces limitations in terms of resources, training, and access to advanced technology [8, 9]. In practical terms, the findings of this study will be of great value to practitioners and administrators in the dental health care sector. By better understanding the relationship between administrative management and service quality, dental practices can implement more effective strategies to improve management, which in turn can lead to more efficient and higher quality care [10]. Improving the service quality in dental offices would not only increase patient satisfaction but could also have a direct impact on health outcomes [11, 12]. The aim of this study was to determine the correlation between administrative management and service quality in dental offices within the context of an upper middle-income country.

## 2. Materials and methods

The research is applied, with an observational, cross-sectional, and exploratory design. The sample was composed of 100 patients treated in dental offices in the city of Andahuaylas, Peru. The sample size was calculated using the EpiDat version 4.2 software to detect a correlation coefficient of 0.276, with a 95% confidence level and 80% power. The recruitment period spanned from August 7, 2023, to September 30, 2023. The sampling was non-probabilistic for convenience. The selection criteria for participants were based on the geographical accessibility of dental offices and their availability to participate in the study. To mitigate potential availability bias in a non-probabilistic sample, several strategies were implemented: participants were recruited from various dental offices in different locations to complete the sample, flexible scheduling accommodated different availabilities, and broad inclusion criteria minimized exclusions. Where possible, randomized invitations were performed within accessible areas to further reduce selection bias. To mitigate response bias, the questionnaire was conducted in a comfortable and private setting at the end of their appointment, ensuring anonymity. Throughout the study, attention was given to ensure the integrity and accuracy of the data collected. After each participant completed the questionnaire, the responses were reviewed for completeness and consistency. Additionally, regular audits of the data entry process were conducted to identify and correct any discrepancies. These measures helped ensure that the recorded information was precise and reliable. Every effort was made to ensure that participants provided complete responses to the questionnaires and minimize the risk of the potential loss of information. Rigorous checks were performed during the data entry process to ensure that no information was lost, including double-checking entries against the original questionnaires. All collected data was regularly backed up to prevent loss due to technical failures, and multiple copies were stored in different secure locations. The research team received thorough training on the importance of accurate and complete data recording, and regular supervision ensured adherence to data collection protocols. To minimize attrition bias, participants were provided with a dental hygiene kit as an incentive.

### 2.1. Questionnaires

In this research, the survey method was used. The data collection instruments were two questionnaires. The administrative management questionnaire, originally proposed by George R. Terry [3] and consisting of 14 closed-ended questions on a nominal scale, was validated for Peruvian population by Pletickosich [13]. The reliability of this instrument, assessed with Cronbach’s Alpha was 89.8%.

The service quality questionnaire, derived from the ServQual tool, and developed by Parasuraman, A.; Zeithaml, V. and Berry, L. [14] consisted of 20 closed-ended questions. This instrument was also validated for the Peruvian population [15]. The reliability, assessed with Cronbach’s Alpha was 82.2%.

Each patient was informed about the purpose and importance of our research and was asked to voluntarily participate in the study. They were required to sign an informed written consent form, authorizing us to use and record their information for the study.

### 2.2. Statistical analysis

The information collected was entered into a Microsoft 365 MS Excel spreadsheet and subsequently subjected to analysis using the STATA SE 18.0. For categorical variables, absolute and relative frequencies were calculated.

The total score in the evaluation of administrative management and service quality was estimated. The Shapiro-French and Doornik-Hansen tests were performed to assess whether the distribution of the data followed a univariate and multivariate normal distribution, respectively, as part of the test of the assumptions for the selection of the statistical test. The results indicated that the score in the evaluation of administrative management meets the normality assumption (univariate normality, p = 0.170; bivariate normality, p = 0.241), while the service quality shows significant deviations from normality (p = 0.044). Therefore, an initial Spearman correlation analysis with corresponding confidence intervals was performed between the scores of the administrative management and service quality dimensions. We consider administrative management as the dependent variable and service quality as the independent variable. To explore the effect of administrative management on the service quality score, a linear regression was performed. To explore the effect of administrative management on the service quality score, a linear regression was performed. Additionally, we evaluated the effect of each dimension of administrative management on the service quality score. Prior to this, the assumptions of linearity, independence of errors, homoscedasticity, and normality of residuals were verified. The analysis was conducted at a 95% confidence level (p < 0.05). Finally, a bidirectional scatter plot of the administrative management and service quality scores was created.

### 2.3. Ethical considerations

The Research Ethics Committee (CIEI) of Cesar Vallejo University reviewed and issued the approval opinion for the execution of this research through the Research Ethics Committee Statement of the School of Stomatology No. 032-2023-/UCV/P.

## 3. Results

### 3.1. Demographic characteristics and other qualities of the patients in a dental context

The Table 1 provides a detailed overview of the demographic characteristics and other qualities of the patients in the context. In terms of gender, there is a relatively even distribution, although slightly skewed toward women, who account for 57% of patients.

**Table 1:** Characteristics of patients surveyed in dental offices within the context of an upper middle-income country.

Characteristics		n (%)
Sex		
	Female	57 (57.0)
	Male	43 (43.0)
Age		
	25 to 35 years old	6 (6.0)
	36 to 45 years old	91 (91.0)
	46 to 55 years old	2 (2.0)
	56 to 65 years	1 (1.0)
Type of health facility		
	Small dental office	99 (99.0)
	Large dental office	1 (1.0)
Time as client		
	<1year	64 (64.0)
	From 1 to 5 years old	32 (32.0)
	From 6 to 10 years old	3 (3.0)
	From 11 years and older	1 (1.0)

In terms of age, there is a marked predominance of the 36–45-year age group, which constitutes 91% of patients. This concentration could indicate that people in this age bracket are more likely to have dental concerns that require regular care, or it could reflect the demographics of the local community.

Finally, most patients (64%) have been receiving treatment for less than one year, indicating a high turnover of new patients. Thirty-two percent have been in treatment for 1 to 5 years, while only a small fraction have been in treatment for 6 to 10 years (3%) or more than 11 years (1%).

### 3.2. Correlation analysis between administrative management and service quality

The Table 2 shows a Spearman correlation analysis between administrative management and service quality, as well as between their different dimensions. Each coefficient represents the degree of correlation between these variables, with a p-value of less than 0.001 in all correlations, indicating high statistical significance.

**Table 2:** Correlation matrix between service quality and administrative management dimensions.

Service quality	Administrative management								
	Planning		Organizing		Directing			Controlling	
	rho	CI 95%	rho	CI 95%		rho	CI 95%	rho	CI 95%
**Tangible elements**	0.42	0.23–0.61	0.68	0.54–0.82		0.62	0.47–0.76	0.58	0.42–0.74
**Reliability**	0.42	0.23–0.61	0.57	0.42–0.72		0.50	0.32–0.69	0.56	0.39–0.73
**Responsiveness**	0.44	0.27–0.61	0.60	0.46–0.74		0.54	0.38–0.70	0.57	0.41–0.73
**Security**	0.57	0.41–0.73	0.66	0.53–0.79		0.64	0.50–0.78	0.62	0.47–0.77
**Empathy**	0.55	0.39–0.71	0.65	0.51–0.78		0.61	0.46–0.76	0.52	0.36–0.68

^a^ Statistical test: Spearman’s correlation. The confidence intervals were calculated using the bootstrap technique with 1000 replications.

^b^ The overall Spearman’s rho between Service quality and administrative management is 0.79 (CI 95% 0.58–0.82).

^c^ The p-value for all correlations was <0.001.

The overall Spearman’s rho is 0.79 (CI 95% [0.58–0.82]), indicating a moderately strong and positive correlation.

In the tangible elements of service quality dimension, it is observed that organizing has the strongest correlation (rho = 0.68, CI 95% [0.54–0.82]), suggesting that effective organizational management is closely related to better tangible elements in service quality. planning, directing and controlling also show moderate positive relationships.

Regarding reliability, all dimensions of administrative management show moderate positive correlations, with organizing (rho = 0.57, CI 95% [0.42–0.72])) and controlling (rho = 0.56, ci 95% [0.39–0.73]) being slightly more influential.

Responsiveness shows a similar trend, with all dimensions of administrative management correlating positively to a moderate degree, indicating that improvements in management could lead to greater responsiveness.

As for security, the coefficient is particularly high for organizing (rho = 0.66, CI 95% [0.53–0.79]) and directing (rho = 0.64, CI 95% [0.50–0.78]), suggesting that these areas are crucial in influencing the perception of safety in the services offered.

Finally, in the empathy dimension, organization (rho = 0.65, CI 95% [0.51–0.78]) and directing (rho = 0.61, CI 95% [0.46–0.76]) again stand out, indicating that these areas of administrative management have a significant impact on perceived empathy in services.

The analysis reveals a positive and significant relationship between administrative management and service quality, as well as between its dimensions, highlighting the importance of effective management to improve the quality of the services offered.

### 3.3. Regression analysis between administrative management and service quality

In the population of patients attending dental offices in Andahuaylas, as the administrative management score increases by one point, the average Service quality score increases by 1.02 points; with a 95% confidence interval of 0.86 to 1.17. This result is statistically significant ([p-value<0.001]) (Table 3).

**Table 3:** Linear regression on the effect of administrative management on service quality.

Administrative management Score		Service quality Score				Service quality Score			
		Coef.	95% CI		p	Coef.	95% CI		p
Administrative Management		1.02	0.86	1.17	< 0.001				
Administrative management Dimensions									
	Planning					0.77	0.06	1.49	0.034
	Organizing					2.11	1.17	3.06	<0.001
	Directing					-0.10	-1.07	0.86	0.830
	Controlling					1.58	0.12	3.05	0.034

^a^ Statistical test: Linear regression

^b^ Negative coefficients correspond to the increase (positive coefficients) or decrease (negative coefficients) in the Service Quality Score.

The coefficient of 0.77 shows a moderately positive relationship with service quality. The 95% confidence interval of 0.06 to 1.49, and the p-value of 0.034 indicates that the relationship is statistically significant, although less strong than administrative management as a whole (Table 3).

The coefficient of 2.11 is the highest, indicating a strong positive relationship with service quality. The 95% CI [1.17, 3.06] and p-value < 0.001 confirm the robustness of this relationship (Table 3).

The coefficient of -0.10 suggests a weak negative relationship with service quality, but the 95% CI [-1.07, 0.86] and p-value of 0.830 indicate that this relationship is not statistically significant (Table 3).

A coefficient of 1.58 indicates a significant positive relationship with service quality. the 95% CI [0.12, 3.05) and p-value of 0.034 support the importance of this dimension (Table 3).

In summary, organizing and controlling are the most critical dimensions of administrative management that affect quality service, while management seems to have a negligible effect.

**Fig 1:**
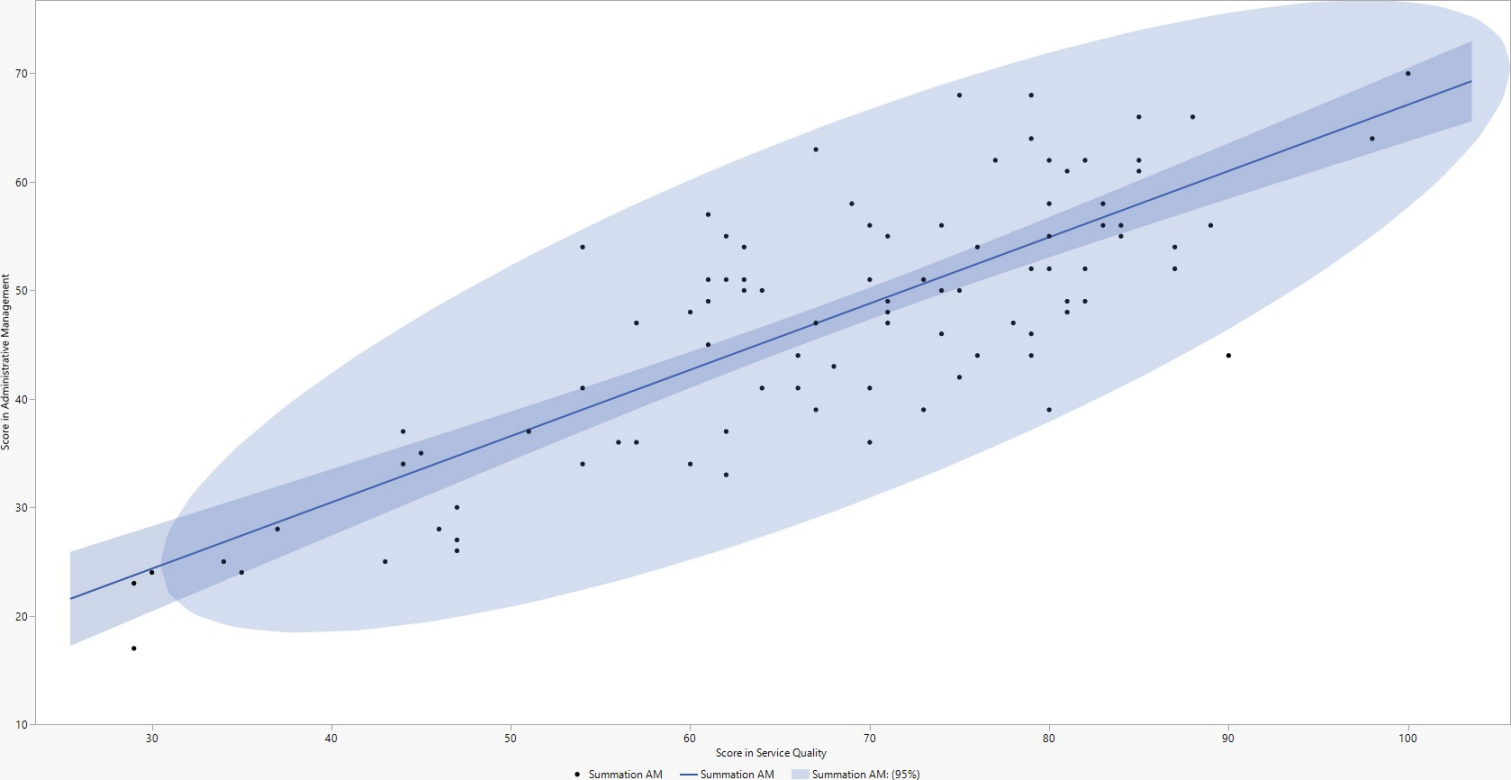
Bidirectional scatter plot of administrative management and service quality scores.

The regression line through the set of points indicates the central tendency of the relationship between the two variables, showing a positive trend: as the administrative management score increases, the service quality score also tends to increase (Fig 1). The shaded band around the regression line represents the 95% confidence interval, estimating where the true values of the regression line are expected to fall for the population. The Fig 1 shows no extreme outliers, and the dispersion of points around the regression line appears uniform, indicating a consistent relationship across different score ranges. The slope of the regression line is moderate, suggesting a moderate relationship between the variables. Overall, the graph suggests a positive correlation between administrative management and quality of care in this data set, indicating that administrative management practices may impact the service quality received.

## 4. Discussion

In our study, carried out in the dental offices within the context of an upper middle-income country, we found a significant, although moderately strong, relationship between administrative management and service quality. This relationship was evidenced by a Spearman correlation coefficient (rho) of 0.79. This result indicates that improvements in administrative management, such as more effective planning, more structured organization, and tighter control, are correlated with higher perceived service quality in these practices. That is, as practices improve in these management areas, the quality of their services also tend to improve.

In addition, the detailed analysis of each of the areas of administrative management revealed that all of them have a positive impact on service quality. Controlling and organizing showed a high correlation with service quality, with values of 1.58 and 2.11 respectively. This suggests that effective control and organization are critical components for improving service quality in dental offices. On the other hand, planning also had a positive relationship with service quality, although to a more moderate degree. These findings further show that, while leadership is important, it may not be perceived as crucial as organizing, controlling, and planning in determining service quality in a dental setting.

Our study demonstrates that administrative management plays a significant role in the service quality offered in dental offices. Although the relationship is not extremely strong, it is clearly positive, indicating that improvements in management can lead to improvements in service quality. This underlines the importance of good administrative management in the dental sector, especially in terms of organization and control.

When comparing our study with similar research in the field of dental service management, we found significant differences and similarities that enrich the understanding of the topic. First, the study by Kunert et al. in Poland [16] highlights the importance of standardizing procedures to improve service quality, a finding that aligns with our study. The perceived need for quality management and the implementation of standardized procedures are consistent with our observation that more effective administrative management leads to better service quality. On the other hand, Lopez et al. in Brazil [17] used methodologies such as Donabedian and Servperf to assess functional quality from the patient’s perspective. Although our study did not use these specific models, the correlation we found between management and service quality supports the idea that improvements in management can positively influence patients’ perception of quality, like the results of Lopez et al. The study by Obadan et al. in the United States [7] highlights a lack of patient focus and a failure to comply with international standardization norms, a critical point that our study also recognizes as important. The need for more patient focus and standardization is consistent with our finding that effective management can improve service quality. Fabian et al. in Peru [18] found a high perception of quality and satisfaction among patients, which reinforces our finding that good administrative management translates into higher service quality. The direct and significant relationship they observed between management and patient satisfaction is further evidence of the importance of effective management. Montalvo et al. [19] also in Peru, highlighted the role of technology in information management and communication to improve the quality of dental services. This complements our results, suggesting that the inclusion of computer technologies in management can be a crucial factor in improving service quality. Finally, Manrique et al. in Peru [12] highlighted that, despite a mostly neutral or negative perception of quality, treatments are mostly adhered to clinical protocols. This suggests that while adherence to protocols is high, there is room for improving the perception of quality through better management, a point that is echoed in our results.

Although our study revealed positive relationships between various aspects of management and service quality in dental offices, it is important to recognize that some of these correlations were moderate or even low. This situation suggests that the measurement of service quality could benefit from a broader or more detailed approach. For example, aspects such as service efficiency and client satisfaction could be incorporated or further analysed in future studies to obtain a more complete picture.

To address another potential limitation of our study, we acknowledge the possibility of selection bias due to the use of non-probabilistic sampling, which may limit the generalizability of the findings. Additionally, there is the potential confounding effect of unmeasured variables such as patient demographics, socioeconomic status, and prior experiences with dental care.

However, it is crucial to emphasize that these limitations in the magnitude of the correlations in no way invalidate the findings of our research. On the contrary, the study provides valuable information into the influence of administrative management on service quality in the dental setting. The identification of a positive relationship, even if moderate, is significant, as it highlights the importance of administrative management as an influential factor in the quality of dental services.

In addition, these results open avenues for future research. For example, specific aspects of management that have a more direct or significant impact on service quality could be further explored. This could include more detailed studies on how the training and professional development of administrative staff, the implementation of quality management systems, or the adoption of advanced technologies contribute to improved patient experience and clinical outcomes.

Despite limitations in terms of the strength of some correlations, our study constitutes an important contribution to the understanding of management in the dental sector and establishes a solid foundation for future research in this field.

One of the main strengths of our study lies in its foundation in a solid theoretical framework, which provided a coherent and structured basis for the research. This theoretical framework not only guided the formulation of hypotheses and research objectives, but also facilitated the interpretation of the results in the broader context of administrative management and service quality in dentistry. In addition, the methodological design we employed was rigorous and carefully planned, which contributed to the validity and reliability of our findings. Although the sample of 100 patients was not probabilistically selected, clear inclusion and exclusion criteria were established to ensure that participants were representative of the demographic group of interest. This methodology allowed for structured and systematic data collection, crucial to the quality of the research.

Another strength of the study is the high confidence level of 95% in the presentation of the results. This high statistical confidence underlines the reliability of our findings and reinforces the credibility of the research. In addition, the use of Spearman’s correlation coefficient to analyse the relationship between administrative management and service quality was a sound methodological choice. This coefficient is particularly suitable for data that do not follow a normal distribution, which is common in behavioural science studies, and provided a robust, nonparametric measure of the correlation between the variables of interest. Complementarily, the analysis resulting from simple linear and multivariate regression provides a deep and detailed understanding of how different aspects of administrative management affect the Service quality in dental offices.

Taken together, these methodological and theoretical strengths ensure that, despite the inherent limitations of any research, the results of our study are reliable and constitute a significant contribution to existing knowledge about dental practice management and its impact on service quality. These aspects strengthen confidence in the findings and recommendations derived from the study, providing a solid foundation for future research and practice in the field.

Despite the limitations we faced, such as the moderate magnitude of some correlations and the non-probabilistic sample size, the results of our study clearly highlight the relevance of effective administrative management in improving service quality in dental offices within the context of an upper middle-income country. This finding is of vital importance, as it underscores the crucial role that management plays in the delivery of high-quality dental health services.

As part of the practical recommendations derived from our findings, we strongly suggest the implementation of advanced management systems in dental practices. This includes process automation, which can lead to greater operational efficiency and reduce human error, as well as staff training in management skills. Training should focus not only on technical competencies, but also on aspects such as decision making, effective communication and leadership, all essential skills for successful management.

In addition, for future research, it is crucial to conduct internal audits on a regular basis. These audits would allow dental practices to continually evaluate and improve their management processes and Service quality. Regular auditing would not only help identify areas for improvement, but would also foster a culture of continuous improvement, essential for maintaining and raising quality standards in dental care.

These actions are not only aligned with the findings of our study but are also based on quality management best practices. By adopting these approaches, dental practices will be able to significantly improve the quality of their services, which in turn can lead to greater patient satisfaction and more successful clinical outcomes. In conclusion, although our study faced certain limitations, the results obtained offer valuable understanding and provide a solid foundation for improving management in the dental sector.

### 4.1. Future scope and significance

The research shows that organization and control in administrative management are key aspects in the perception of service quality. Therefore, it is recommended that these areas be strengthened through the implementation of more efficient management systems, training of administrative personnel, and the adoption of technologies that optimize these processes.

## 5. Conclusion

There is a positive and moderately strong, statistically significant correlation between administrative management and service quality in dental offices within the context of an upper middle-income country. Improving administrative management could, therefore, have a direct impact on the perception of service quality. Different aspects of administrative management have varying degrees of impact on service quality. While organizing and controlling (β = 1.58; 95% CI [0.12; 3.05]; p = 0.034) in administrative management are critical and show a significant positive relationship, directing (β = -0.10; 95% CI [-1.07; 0.86]; p = 0.830) appears to have an insignificant effect. This highlights the importance of focusing on specific areas to improve the effectiveness of administrative management in dental offices.
